# Syndromic Inborn Errors of Immunity in TREC-Newborn Screening: 5-year Experience from the German Screening Program

**DOI:** 10.1007/s10875-026-01995-2

**Published:** 2026-03-14

**Authors:** Lea Graafen, Carsten Speckmann, Shahrzad Bakhtiar, Horst v. Bernuth, Kai Lehmberg, Peter Bader, Ulrich Baumann, Rita Beier, Stephan Borte, Inken Brockow, E. Graham Davies, Maximilian Hartmann, Ursula Holzer, Christian Klemann, Alexandra Y. Kreins, Renate Krüger, Udo Kontny, Hans-Jürgen Laws, Andrea Meinhardt, Henner Morbach, Nora Naumann-Bartsch, Tobias Rothoeft, Dominik T. Schneider, Andre Willasch, Austen Worth, Markus G. Seidel, Michael H. Albert, Stephan Ehl, Fabian Hauck, Manfred Hönig, Ansgar Schulz, Catharina Schuetz, Sujal Ghosh

**Affiliations:** 1 Department of Paediatric Oncology, Haematology and Clinical Immunology, Medical Faculty, Heinrich-Heine- University, University Hospital Düsseldorf, Moorenstraße 5, Düsseldorf, 40225 Germany; 2 Institute for Immunodeficiency, Centre for Chronic Immunodeficiency (CCI), Faculty of Medicine, Medical Centre, University of Freiburg, Freiburg, Germany; 3 Department of Paediatric Haematology, Oncology and Stem Cell Transplantation, Medical Centre, Faculty of Medicine, Children’s Hospital, University of Freiburg, University of Freiburg, Freiburg, Germany; 4 Division for Stem Cell Transplantation and Immunology, Department for Children and Adolescents, University Hospital Frankfurt, Goethe University, Frankfurt Am Main, Germany; 5 Department of Paediatric Respiratory Medicine, Immunology and Critical Care Medicine, Charité - Universitätsmedizin Berlin, corporate member of Freie Universität Berlin, Humboldt-Universität zu Berlin and Berlin Institute of Health (BIH), Berlin, Germany; 6 Labor Berlin Charité -Vivantes, Department of Immunology, Berlin, Germany; 7 Charité - Universitätsmedizin Berlin, corporate member of Freie Universität Berlin, Humboldt-Universität zu Berlin, Berlin Institute of Health (BIH), Berlin-Brandenburg Centre for Regenerative Therapies (BCRT), Berlin, Germany; 8 Division of Paediatric Stem Cell Transplantation and Immunology, Clinic for Paediatric Haematology and Oncology, University Medical Centre Hamburg-Eppendorf, Hamburg, Germany; 9 Department of Paediatric Pulmonology, Allergy and Neonatology, Hannover Medical School, Hannover, Germany; 10 Paediatric Haematology and Oncology, Hannover Medical School, Hannover, Germany; 11 Jeffrey Modell Diagnostic and Research Centre for Primary Immunodeficiency Diseases, Immune Deficiency Centre Leipzig, Hospital St. Georg, Leipzig, Germany; 12 Screening Centre, Bavarian Health and Food Safety Authority (LGL), Oberschleissheim, Germany; 13 Department of Immunology and Gene Therapy, Great Ormond Street Hospital for Children, London, UK; 14 University Children’s Hospital, Eberhard Karls University, Tübingen, Germany; 15 Department for Paediatric Immunology, Rheumatology & Infectiology, Hospital for Children and Adolescents, University of Leipzig, Leipzig, Germany; 16 Infection, Immunity and Inflammation Research & Teaching Department, Great Ormond Street Institute of Child Health, University College London, London, UK; 17 Division of Paediatric Haematology, Oncology and Stem Cell Transplantation, Medical Faculty, RWTH Aachen University, Aachen, Germany; 18 Paediatric Haematology, Oncology and Immunodeficiencies, University Children’s Hospital Giessen, Giessen, Germany; 19 Department of Paediatrics, University Hospital of Würzburg, Würzburg, Germany; 20 Division of Paediatric Haematology and Oncology, Department of Paediatrics, University Hospital Erlangen, Erlangen, Germany; 21 Department of Paediatrics, Paediatric Intensive Care Medicine, Catholic Hospital Bochum, Ruhr-University of Bochum, Bochum, Germany; 22 Clinic of Paediatrics, Municipal Hospital Dortmund, Dortmund, Germany; 23 Research Unit for Paediatric Haematology and Immunology, Division of Paediatric Haemato-Oncology, Department of Paediatric and Adolescent Medicine, Medical University Graz, Graz, Austria; 24 Department of Paediatrics, Dr. von Hauner Children’s Hospital, University Hospital, Ludwig-Maximilians- Universität München, Munich, Germany; 25 Department of Paediatrics, University Medical Centre Ulm, Ulm, Germany; 26 Department of Paediatrics, Medizinische Fakultät Carl Gustav Carus, Technische Universität Dresden, Fetscherstraße 74, Dresden, 01307 Germany; 27 German Centre for Child and Adolescent Health (DZKJ), Partner Site Leipzig/Dresden, Dresden, Germany

**Keywords:** Newborn screening, T-cell receptor excision circles, SCID, Syndromic inborn errors of immunity, T-cell lymphocytopenia, Thymic deficiency

## Abstract

TREC-NBS identifies patients with inborn errors of immunity (IEI) and syndromic features, but uncertainty remains regarding their immunological management. To address this, syndromic patients detected by TREC-NBS in Germany between August 2019 and April 2024 were systematically analyzed, including phenotype, treatment, and outcomes. National registries were screened, and data were completed by the treating centres. A total of 77 syndromic patients were identified, with 22 different gene defects found in 72 individuals (93.5%). Primary thymic deficiency was present in 64% (49/77), most commonly due to 22q11.2 deletion syndrome (62%). Common clinical features included congenital heart disease (57%), facial/skeletal abnormalities (53%), and neurological symptoms (36%). Definitive treatments were provided promptly in eligible patients, including 6 thymus transplants and 6 hematopoietic stem cell transplants (HSCT). A watch-and-wait approach was applied to the remaining patients, with 34% (22/65) receiving prophylactic treatment. Recovery of CD3 + T-cell counts was limited to a minority. Overall survival was 89%, with a median follow-up of 32 months (range 0.5–60). To conclude, this is the first comprehensive study of syndromic IEI patients identified through TREC-NBS. The findings show that the German healthcare system enables both early prophylactic care and timely access to definitive therapies. Moving forward, interdisciplinary collaboration will be key to developing evidence-based management guidelines for this challenging patient group.

**Supplementary Information:**

The online version contains supplementary material available at 10.1007/s10875-026-01995-2.

## Introduction

Screening for T-cell receptor excision circles in newborn screening (TREC-NBS) allows for early detection of severe combined immunodeficiency (SCID) patients, paving the way for timely definitive treatment, better survival and beneficial immune-related outcomes [1]. Beyond this remarkable benefit for the management of SCID patients, TREC-screening also allows for the detection of non-SCID conditions with T-cell impairment, including a variety of genetic syndromes [2, 3]. 

These infants with inborn errors of immunity (IEI) and syndromic features constitute a highly heterogenous group of patients, with the majority of them not requiring immunological corrective procedures, such as haematopoietic stem cell transplantation (HSCT). Some even display spontaneous, although often partial, immunological recovery over time. Nevertheless, even in the absence of a need for corrective treatment, the timely implementation of protective measures is crucial when clinically relevant immunological impairment is present [2]. These syndromic diseases may therefore be regarded as “bonus” targets of the TREC-NBS, rather than “false positives”, whose clinical management is complicated by frequent multi-organ involvement of the diseases.

In Germany, TREC-NBS was added to the national NBS-program in August 2019 and constitutes the largest TREC-screening program in Europe since then. As expected, a first analysis after 2.5 years revealed that 55% (46/88) of newborns with abnormal TREC copy numbers were diagnosed with an IEI with syndromic features [4]. To date, a structured approach to describe the essential characteristics, the management and outcomes of this cohort systematically, has not been conducted in any existing NBS-program. Yet, this is essential to assess the clinical implications and outcomes of these screening-positives, and to establish evidence-based treatment guidelines in the future.

Hence, in this work we present the first in-depth evaluation of syndromic neonates with IEI detected by TREC-NBS through a comprehensive analysis of the German national registry data. Our findings provide significant insights into the clinical presentation, monitoring, and applied treatment strategies applied in this cohort.

## Methods

In Germany, TREC screening, as part of the nationwide NBS program, is overseen by the Paediatric Directive (*Kinderrichtlinie*) of the Federal Joint Committee (*Gemeinsamer Bundesausschuss*, G-BA) [5, 6]. The detailed algorithm of the German TREC-NBS program has been published earlier [4]. Patients with absent TREC levels on their first screening card or confirmed T-cell lymphocytopenia (TCL) following repeatedly abnormal TREC-results are referred to one of the specialized regional Combined Immunodeficiency Centres, i.e., “CID-Centres,” as defined by the responsible medical societies. Confirmatory testing of an abnormal but non-urgent TREC-result (i.e., reduced but not absent TREC) may be conducted by a designated local “CID-Clinic” as well. The latter fulfil defined structural quality criteria, but do not require as many resources, including a stem cell transplant unit, as compared to “CID-Centres.” [7].

NBS will only be performed if the legal guardians have provided their written informed consent and authorized data transfer to the screening laboratory [5]. At present, there is no centralized database for nationwide tracking of NBS results in Germany. However, as part of a plausibility assessment, the German Society for Newborn Screening, i.e., *Deutsche Gesellschaft für Neugeborenenscreening* or DGNS, tracks the number of patients with a positive NBS, including those with a positive TREC-result, and publishes the results in yearly intervals in the DGNS report [8]. Moreover, the German Working Group for Paediatric Immunology (*Arbeitsgemeinschaft Pädiatrische Immunologie* or API) performs surveys at six-month intervals collecting data from all CID-Clinics and Centres, who report all patients identified by TREC-screening and on SCID patients, who were possibly missed by the TREC-NBS. In addition to these registry-derived patient data, the analysis at hand covers datasets by each referral centre, i.e., both CID-Clinics or Centres, including information on the medical history, the clinical phenotype, detailed immunological investigations, medical treatments, and the psychomotor development. Immunological findings following either HSCT or thymus transplantation were excluded from the final analysis. Specific diagnostic procedures applied by the individual centre were not systematically evaluated.

Our analysis reports all patients with IEI and syndromic features detected by TREC-NBS in Germany in the time period from August 2019 until April 2024. The patients were classified as ‘syndromic’ (i.e., a constellation of symptoms) either according to the 2024 Human Inborn Errors Classification of the International Union of Immunological Societies, or on the basis of their individual or disease-associated clinical phenotypes [9]. 

Evaluation and statistical analyses were performed using the Graph Pad Prism^®^ Software. Differences between groups were calculated with unpaired student’s *t*-test.

## Results

### General Screening Results and Patients’ Characteristics

Between August 2019 and April 2024, TREC-NBS was performed on approximately 3.1 million newborns throughout Germany. Among these, 77 newborns with syndromic IEI were identified, resulting in an incidence of approximately 1:40,000 newborns. In 44% (31/70) of these neonates, TREC copy numbers were low, and in 56% (39/70) absent. During the same period 58 neonates were identified with classical SCID, leaky SCID or Omenn Syndrome (unpublished data).

Among patients with IEI and syndromic features, consanguinity was reported in 14% (11/77) of parents. Only 17% (13/77) of patients had a positive family history for haematological or immunological diseases.

**Fig. 1:**
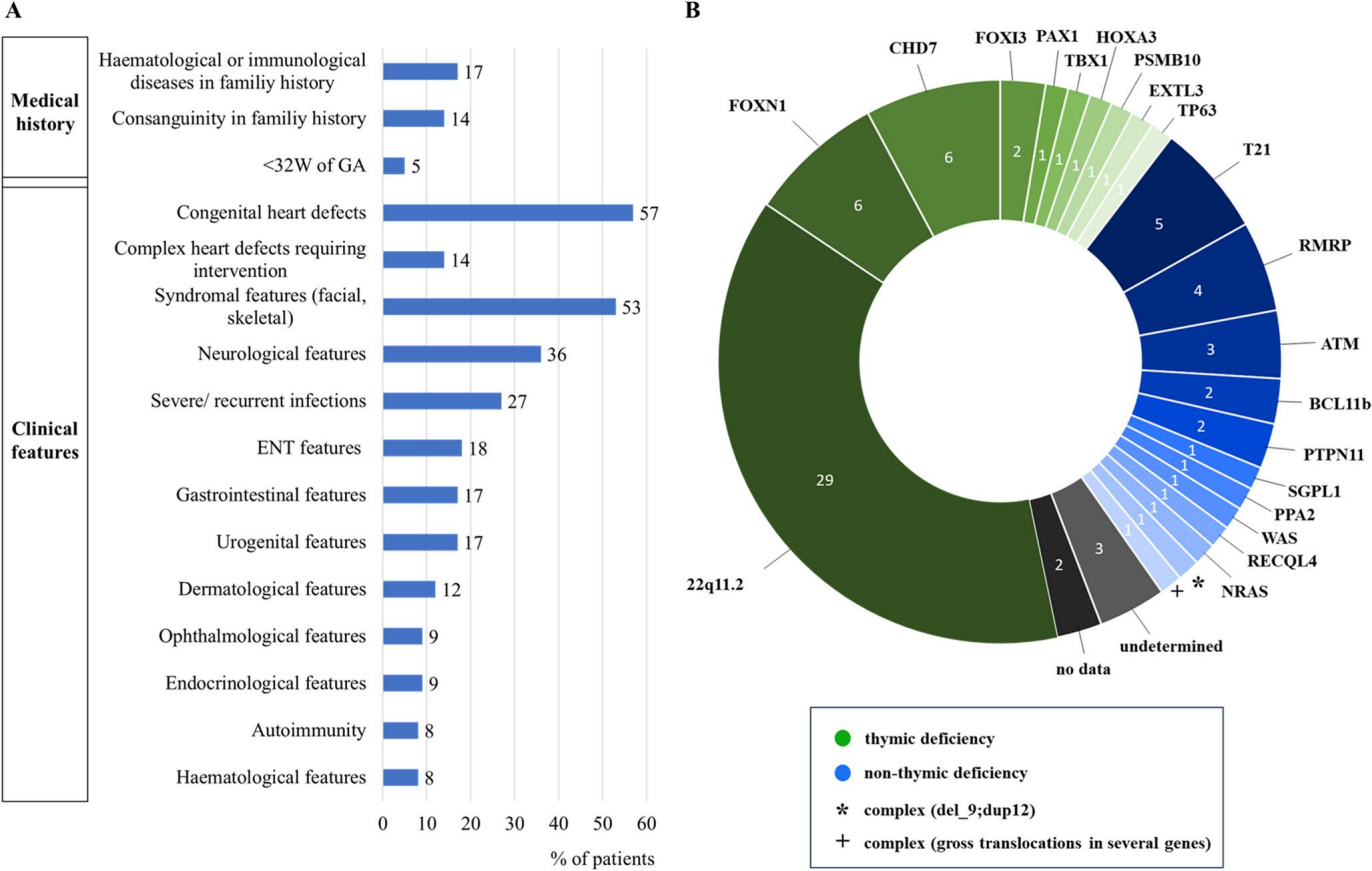
Clinical findings and affected genes in syndromes associated with T-cell lymphocytopenia detected by TREC-NBS in Germany from August 2019 – April 2023. (**A**) Patient clinical characteristics, including medical history and key clinical features (% of all patients, *n* = 77). (**B**) Affected genes in the syndromic patient cohort with the numbers of cases indicated. Green: Thymic deficient syndromes. Blue: Non-Thymic deficient syndromes. Grey: undetermined. Black: no data on genetics available

93.5% of all patients (72/77) were found to have an underlying genetic finding, revealing 22 different genetic syndromes associated with TCL; see Fig. 1. In only 4% (3/77) genetic results were inconclusive. For the remaining 2 patients, no data on genetic findings were available for our analysis. Overall, variants in genes associated with thymic deficiency (TD: 64%; 49/77) were considerably more often identified in this cohort than variants associated with non-thymic deficiency (NTD: 36%; 28/77); see Fig. 1. *22q11.2* deletion syndrome (*22q11.2DS*) was by far the most frequent genetic diagnosis and was found in 38% (29/77) of the screening-positive neonates with identified IEI and syndromic features. Other genetic findings concerned *FOXN1* haploinsufficiency and compound-heterozygous *FOXN1* mutations (8%; 6/77), as well as mutations in *CHD7* (8%; 6/77), trisomy of chromosome 21 (T21: 6.5%; 5/77) and mutations in *RMRP* (5%; 4/77). Nine neonates had mutations in the following genes: *ATM* (4%; 3/77), *FOXI3*, *BCL11B* and *PTPN11* (2.5% each, or 2/77). Other singular genetic findings accounted for 17% (13/77) of the cohort; see Fig. 1.

### Clinical Characteristics

With a median follow-up time of 32 months (range 0.5–60), the most frequently reported characteristics were congenital heart defects (57%; 44/77), facial or skeletal findings (53%; 41/77), and neurological features (36%; 28/77); see suppl. Material 1. Minor cardiac pathologies such as patent foramen ovale (48%; 21/44) or ventricular septal defects (25%; 11/44) were common. More complex heart defects were described in a quarter of patients (25%; 11/44). As expected, the majority of those (63%; 7/11) had a TD as part of *22q11.2DS*, a condition in which heart defects are often reported.

The most frequently documented neurological sign was developmental delay. Considering the limitations in evaluating the development in young infants, developmental deviations comprised 50% (14/28) of neurological findings. Other neurological features varied and included structural abnormalities of the central nervous system (18%; 5/28), muscular hypotonia, hypotonia of the pharynx, and feeding disorders (each 11%; 3/28). Aside from this global physician assessment, no standardized developmental scale evaluation was included in this survey.

Severe or recurrent infections were documented in 27% (21/77) of all patients. Other organ manifestations were of gastrointestinal (17%; 13/77), urogenital (17%; 13/77), dermatological (12%; 9/77), ophthalmological (9%; 7/77), endocrinological (9%; 7/77), haematological (8%; 6/77) and autoimmune origin (8%; 6/77); see suppl. Material 1 for clinical features in individual patients.

## Immunological Findings

### Lymphocyte Subsets

**Fig. 2:**
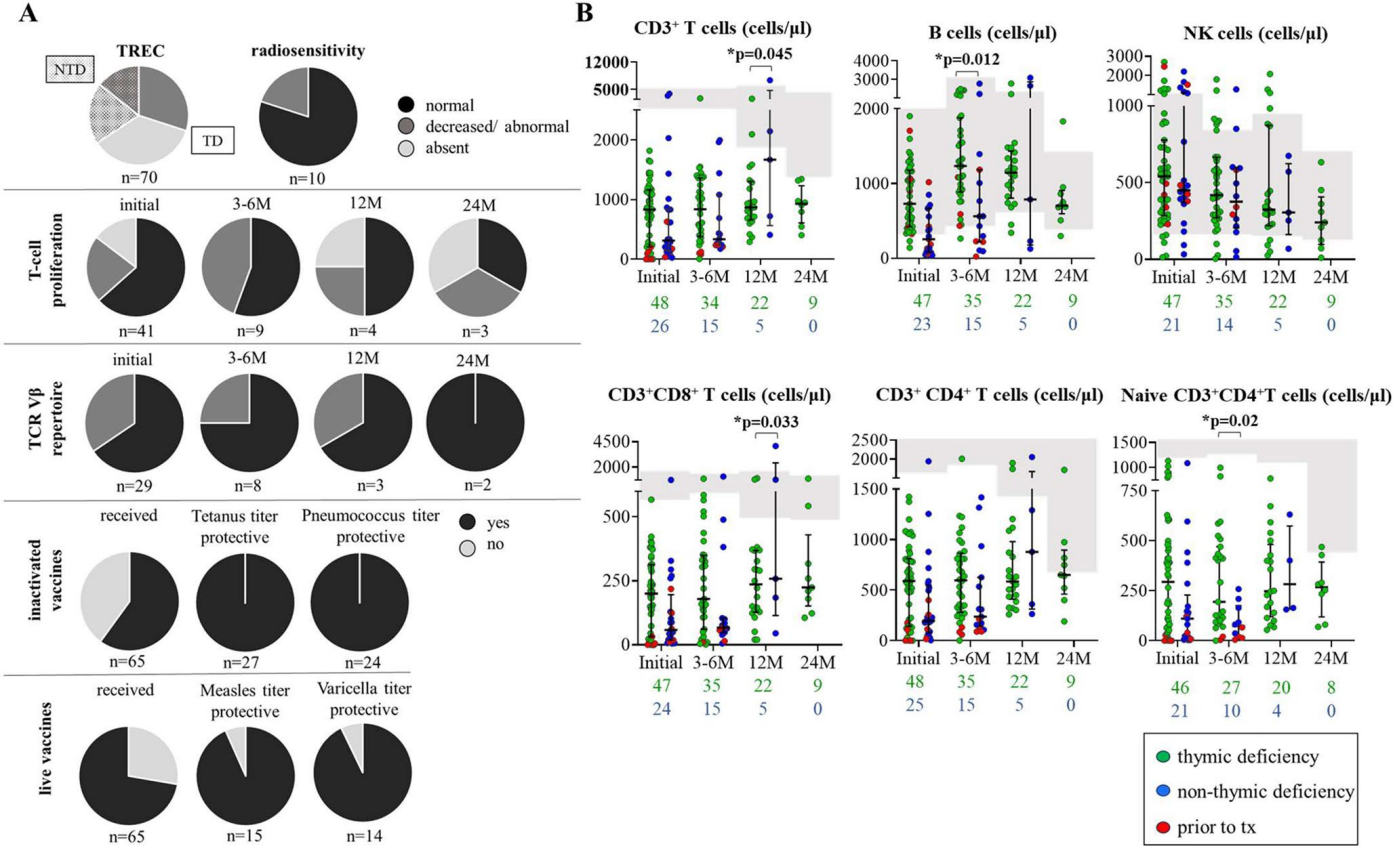
Immunological findings in the syndromic patient’s cohort. (**A**) Information on TREC copy numbers, radiosensitivity testing, T-cell proliferation, TCR Vβ repertoire and immunization responses. (**B**) Cell counts of thymic deficient (green) and non-thymic deficient (blue) syndromic patients at different time points: initial screening, at 3–6, 12 and 24 months of age. Patients who underwent HSCT or thymus transplantation at a later timepoint are indicated in red within their group. Approximate age-dependent paediatric reference values according to Shearer et al. (2003) in grey. Cell counts of total lymphocytes, total T cells, CD8 + and CD4 + T cells, naïve CD4 + T cell, B cell and NK cells, as well as available patient numbers at each time point (below the graphs). Cell counts after transplantation not included. Black bars indicate median and interquartile range. Student’s t-test was applied to assess significance

Cell counts including CD3^+^ T cells, CD20^+^/19^+^ B-cells and CD3^−^CD56^+^/16^+^ NK cells, as well as T-cell subsets, i.e., CD4^+^ T-helper cells, CD4^+^CD45RA naïve T-helper cells, CD8^+^ cytotoxic T cells were documented at 4 different time points: At first screening, at 3–6, 12 and 24 months of age; see Fig. 2. CD3^+^ T cells and T-cell subsets were significantly lower in patients with absent TREC-results compared to patients with low TREC-results at initial presentation and at 3–6 M of age (for CD3^+^ T cells: p = < 0.0001 vs. *p* = 0.003 respectively; see supplementary Fig. 1). There was no significant difference in initial T-cell numbers comparing TD and NTD patients, i.e., TD: median 832 (min-max 0–1818), NTD: median 336 (min-max 27–3938) CD3^+^ T cells/µl. However, CD3^+^ T-cells and CD8^+^ T-cell subsets significantly differed between these two groups at 12 months of follow-up (*p* = 0.045 vs. *p* = 0.033 respectively) and were higher in NTD patients. At the same time, NTD patients had a significantly lower naïve CD4 + T-cell count at 3–6 M of age compared to TD patients (*p* = 0.02). Additional abnormalities in B- and NK-cell numbers were found in a proportion of the cohort, but did not significantly differ between TD and NTD patients; see Fig. 2.

8 patients had a very low initial CD3^+^ T-cell count of < 50 cells/µl (ID3, ID14, ID15, ID20, ID26, ID36, ID51, ID52). Five of them had a TD, including 4 patients diagnosed with congenital athymia. 3 of these patients received thymus transplantation: 2 with *22q11.2DS* and 1 with *CHD7* haploinsufficiency. The fourth athymic patient with *22q11.2DS* died due to non-immunological complications before thymus transplantation could be offered. The fifth TD patient was diagnosed with compound heterozygous *FOXN1* deficiency, and T-cell counts improved over time. A group of 3 patients with NTD conditions was comprised of one child with cartilage hair hypoplasia, i.e., CHH, mutations in *RMRP*, who underwent HSCT, while the other 2, i.e., 1 patient with T21 and 1 with a mutation in *SGLP1*, died of non-immunological causes; see **s**upplementary Material 1.

Overall, only 5 patients demonstrated complete recovery of CD3^+^ T-cell counts during the follow-up period (ID 56, ID 28, ID 46, ID 59, ID 74). Two of these patients had a TD: one of them due to *FOXN1-*haploinsufficiency and the other one due to a *TP63*-gene mutation. The third patient was diagnosed with NTD and with a mutation affecting the *BCL11B*-gene. In the fourth patient the genetic condition remained unidentified. Genetic data were not available for one individual infant.

### T-cell proliferation, TCR Vβ Repertoire and Radiosensitivity Testing

T-cell proliferation and T-cell receptor (TCR) Vβ repertoire were studied in 58% (45/77) and 45% (35/77) of patients, respectively, at any timepoint; see Fig. 2. At inital measurement, T-cell proliferation was normal in 63% (26/41), impaired in 22% (9/41), and absent in 6% (6/41) of the tested individuals. At the same time, diversity of the TCR Vβ repertoire was normal in 66% (19/29) and abnormal in 34% (10/29). Of the 10 patients who underwent radiosensitivity testing at any timepoint, 2 (20%) had abnormal results; see Fig. 2. Available functional assay results were consistent with the molecular findings in all neonates with an established genetic diagnosis.

### Vaccine Responses

Within the cohort of conservatively treated patients, 60% (39/65) were vaccinated with inactivated vaccines and 28% (18/65) received live vaccinations; see Fig. 2. In accordance with the national recommendation for vaccinations in immunodeficient patients, all of the patients who received live immunizations had CD3^+^ T-cell counts > 500/µl and CD4^+^ T-cell counts > 200/µl [10]. T-cell proliferation was tested normal in 61% of these 18 individuals (11/18), and titres against inactivated vaccines were protective in all but one child (94%, 17/18). Finally, no complications following the administration of vaccinations were reported.

The assessment of vaccination responses in all vaccinated patients revealed protective titres in all tested individuals for inactivated vaccines, while responses to live vaccinations were protective in all but 2 individuals (ID21, ID73). These 2 patients exhibited mixed responses to live vaccinations showing protective titres against either varicella or measles, but not to both. However, responses to inactivated vaccines had previously been tested as normal.

### Therapeutic Management

Overall, 44% (34/77) of our cohort received prophylactic treatments. The most frequently used drug was cotrimoxazole (42%, 32/77). Other antibiotics were rarely used (5%, 4/77). Further prophylactic treatments included antifungal (35%, 27/77) and antiviral drugs (14%, 11/77) as well as immunoglobulin replacement therapy (IGRT: 27%, 21/77). Immunosuppressive treatments outside the context of transplantation were reported in 6 individuals, although one of these received immunosuppression for autoimmune-haemolytic anaemia post-thymus transplantation despite prior immune reconstitution; see supplementary Material 1 for treatment strategies in individual patients.

Corrective treatments were applied in 16% (12/77) of the patients. Of those, 6 patients with congenital athymia underwent thymus transplantation at a median age of 3.5 months (2–11 months). Another 6 patients with hematopoietic cell intrinsic defects received HSCT at a median age of 7.5 months (2–11 months). All of these patients were alive at the time of last follow-up. There were no patients for whom HSCT or thymus transplantation was indicated but could not be provided due to structural constraints. However, one athymic patient with *22q11.2DS* died following a cardiac procedure before thymus transplantation could be offered (ID20).

### Survival and Outcome

**Fig. 3:**
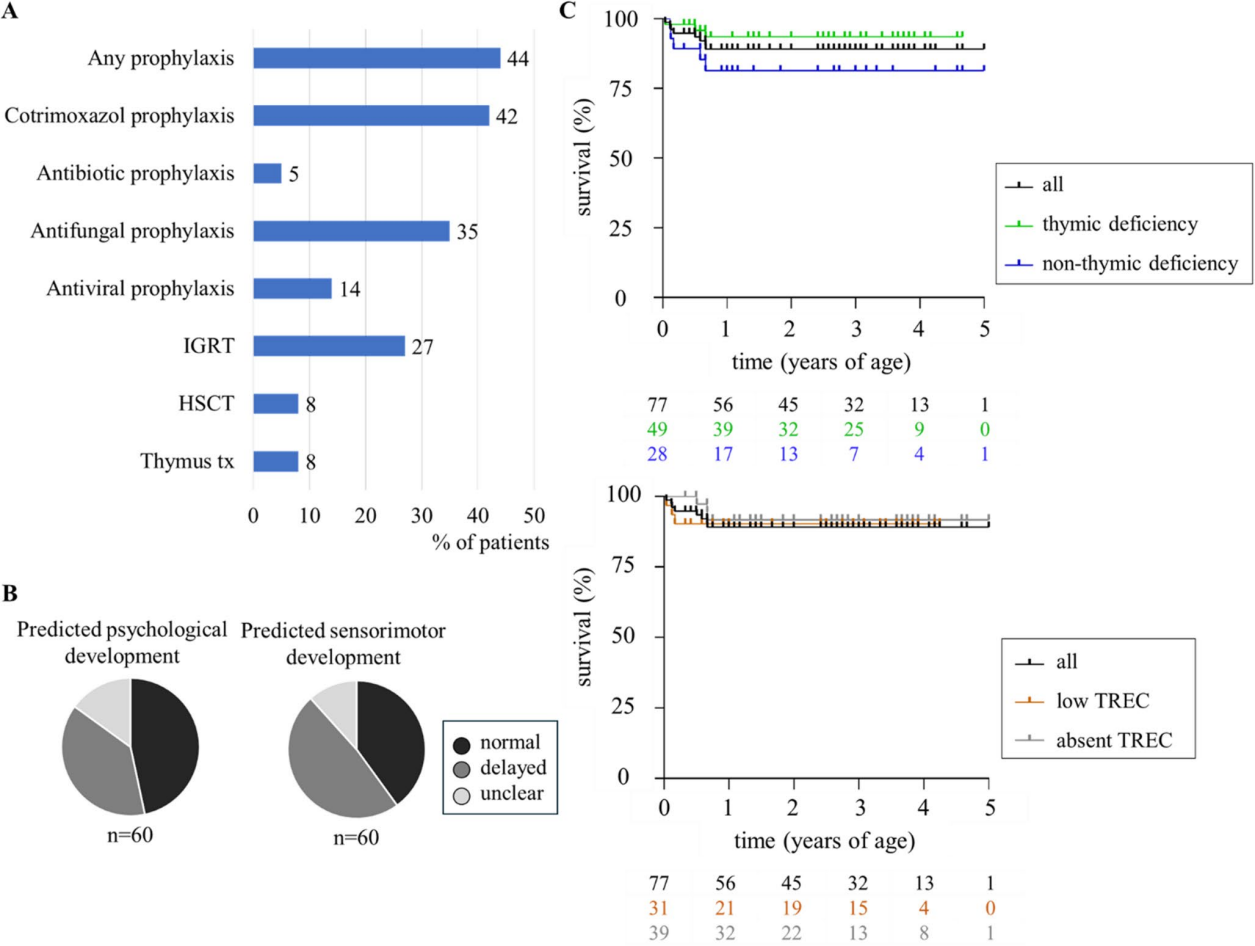
Treatment modalities and outcome at last follow-up. (**A**) Treatments employed in our cohort (% of patients, *n* = 77). (**B**) Predicted psychomotor development by global physician assessment. (**C**) Kaplan-Meier survival curves for all (black), thymic deficient (green) and non-thymic deficient patients (blue), as well as for patients with low TREC-results (brown) and absent TREC-results (grey) on newborn screening, including patients who received transplantation. Cumulative survival probability over time is illustrated. Censoring events are marked by vertical ticks on the curves. Statistical differences between the groups were assessed using the log-rank test

After a median follow-up of 32 months (0.5–60 months), overall survival in our cohort of syndromic IEI patients detected by TREC-NBS was excellent (89%). With survival of 93.5%, the outcome was superior in patients with TD compared to patients with NTD (survival rate of 81%); see Fig. 3. There was no difference in overall survival comparing patients with low TREC-results and patients with absent TREC-results on their NBS. However, 9 patients died at a median age of 4 months (11 days – 8 months); see Fig. 3. Causes of death were mostly of non-immunological origin and more likely related to cardiac or respiratory complications; see suppl. Material 1.

In detail, two patients with *22q11.2DS* died as a consequence of their cardiac defect: 1 patient with cardiac decompensation at 11 days of age (ID43), and 1 patient with a complication post cardiac procedure at 6 months (ID20); see suppl. Material 1. Another patient experienced sudden cardiac death in the context of *PPA2*-associated disease at the age of 1.5 months (ID16). Three patients died due to respiratory failure. Among these, immunological involvement may have contributed to the patient’s demise in one case of *T21* with aspiration pneumonia at the age of 2 months (ID67). The other two patients died at 7 weeks (*SGLP1*, ID14) and 8 months (*T21*, ID15) respectively. One patient with *CHD7* deficiency died of upper airway obstruction due to severe hypotonia of the pharynx at the age of 8 months (ID49). Another patient with an undefined genetic syndrome, experienced rapid progressive neurodegenerative disease and died at the age of 8 months (ID38). Finally, for one patient of our cohort, the cause of and age at death were not documented (ID77).

At last follow-up, the global judgement of a physician in respect of predicted psychomotor development was available for 88% (60/68) of survivors. Psychosocial development was reported as normal in 47% (28/60), as delayed in 38% (23/60) and as uncertain in 12% (9/60); see Fig. 3. Similarly, the predicted sensorimotor development was rated as normal in 40% (24/60), as delayed in 48% (29/60) and as uncertain in 12% (7/60).

## Discussion

This present study provides a comprehensive analysis of the German syndromic patient cohort detected by TREC-NBS since 2019, representing the first study worldwide to focus in detail on this heterogenous and multifaceted cohort.

During the last decades, the TREC-screening has been implemented in a number of countries worldwide, including all US states and 17 European countries (15 national and 2 regional TREC-screening programs) [11–14]. The German program is currently the largest in Europe, achieving 99.1% coverage of 738,819 newborns screened in 2022 [8]. Patients with IEI and syndromic features constitute the majority of patients with inborn TCL detected by TREC-NBS in Germany, similar to findings from other programs worldwide, particularly in the US [2, 13, 15, 16]. However, detailed clinical characterization of this subgroup has been lacking, leaving phenotypes, management decisions, and outcomes insufficiently defined.

In Europe, other TREC-(pilot-) screening programs report varying proportions of syndromic IEI among screening positives, ranging from approx. 18% in France to 60% in Sweden [17–21]. These discrepancies reflect both true differences in disease incidence and program-specific factors such as screening algorithms and TREC cut-off values. Combined TREC/KREC screening, as used in Sweden, Ukraine, and Switzerland, broadens the immunological phenotype detectable to include syndromic conditions [12, 22, 23], whereas integration of next-generation sequencing (NGS) in the Norwegian program enhances the specificity for classical SCID [19]. 

These variations mirror the ongoing debate regarding the early detection of this “bonus target group” of TREC-NBS [3, 24]. Unlike the “primary target group” of typical and atypical SCID, definite treatments such as HSCT or gene therapy are often not applicable in syndromic infants. Moreover, multisystem involvement may complicate treatment decisions; for example, severe neurological impairment can delay or preclude HSCT. Importantly, patients with congenital athymia require thymus transplantation for curative therapy, currently performed at a single European centre [25]. 

A notable disease entity worth discussing is Ataxia Telangiectasia (AT), characterized by mild to severe combined immunodeficiency and cancer predisposition. Most critically, these patients suffer from a profound progressive neurodegeneration, leading to a markedly reduced life expectancy [26]. Despite the immunodeficiency being caused by a cell-intrinsic defect, the use of HSCT for children with severe immunological affection is complicated by the pronounced radiosensitivity and significant drug toxicity associated with AT. For these reasons, an ethical debate on the early detection of AT patients through NBS has emerged [24, 27]. 

Systematic analysis of syndromic IEI detected by TREC-NBS is therefore essential to establish future consensus on their management. The German program thus offers a unique opportunity to characterize this syndromic group in detail. Finally, optimal care requires close interdisciplinary collaboration with immunologists, neurologists, cardiologists, and other professionals.

Our data underscore the importance of continued immunological monitoring and individualized management of syndromic patients identified through TREC-NBS. Although partial immune recovery occurs in some of these cases, complete recovery is exceptional. Preventive measures, including prophylactic drugs, a proper vaccination regime, and, in selected cases, discontinuation of breastfeeding in CMV-positive mothers, remain crucial to prevent life-threatening infections [28]. Laboratory parameters that may inform decision-making regarding a “watch-and-wait” approach versus the initiation of prophylactic treatments include the absolute T-cell counts, T-cell proliferation assay results, and vaccine responses. However, standardized criteria for such decisions are lacking.

Late-onset autoimmunity or malignancy, as well as infection frequency, may be underrecognized in our cohort, reinforcing the importance of long-term follow-up. However, our data clearly demonstrate the effectiveness of early diagnosis and coordinated management of the syndromic patients with TCL, as well as the strength of national referral networks and the value of early therapeutic intervention if necessary [29–31]. Finally, the high diagnostic yield of the German program supports precision-based management for this complex group.

Overall, our results are unique in providing an in-depth analysis of syndromic IEI detected through TREC-NBS, using data from the largest European NBS-program. Profound TCL was demonstrated in most patients, exposing them at a substantial risk for life-threatening infections unless detected and managed early. The German referral structure, combined with well-functioning international collaborations, ensures timely access to both supportive and definitive therapies. Continued longitudinal analysis of national TREC-NBS programs will help improve the management of this heterogenous patient group. In the future, expert consensus guidelines for principles of management are needed.

## Supplementary Information

Below is the link to the electronic supplementary material.

Supplementary Material 1 (XLSX 345 KB)

Supplementary Material 2 (PDF 400 KB)

## Data Availability

No datasets were generated or analysed during the current study.
