# Supplementary material for: Syndromic Inborn Errors of Immunity in TREC-Newborn Screening: 5-year Experience from the German Screening Program
Source: J Clin Immunol. 2026 Mar 14;46(1):35. doi: 10.1007/s10875-026-01995-2 (PMC13031199; doi:10.1007/s10875-026-01995-2)
Supplement: Supplementary file 2 — Supplementary Material 2 (PDF 400 KB) [file 10875_2026_1995_MOESM2_ESM.pdf]

**Supplementary material**

**Submitted to the Journal of Clinical Immunology**

# Syndromic Inborn Errors of Immunity in TREC-Newborn Screening: 5-year experience from the German Screening Program

Lea Graafen, Carsten Speckmann, Shahrzad Bakhtiar, Horst v. Bernuth, Kai Lehmberg, Peter Bader, Ulrich Baumann, Rita Beier, Stephan Borte, Inken Brockow, E. Graham Davies, Maximilian Hartmann, Ursula Holzer, Christian Klemann, Alexandra Y. Kreins, Renate Krüger, Udo Kontny, Hans-Jürgen Laws, Andrea Meinhardt, Henner Morbach, Nora Naumann-Bartsch, Tobias Rothoeft, Dominik T. Schneider, Andre Willasch, Austen Worth, Markus G. Seidel, Michael H. Albert, Stephan Ehl, Fabian Hauck, Manfred Hönig, Ansgar Schulz, Catharina Schuetz*, Sujal Ghosh*

*contributed equally

**Corresponding authors:**

Sujal Ghosh, MD, Department of Paediatric Oncology, Haematology and Clinical Immunology, Medical Faculty, Heinrich-Heine-University, University Hospital Düsseldorf. Moorenstraße 5, 40225 Düsseldorf, Germany. E-mail: sujal.ghosh@med.uni-duesseldorf.de

Catharina Schuetz, MD, Department of Paediatrics, Medizinische Fakultät Carl Gustav Carus, Technische Universität Dresden, Dresden, Germany. Fetscherstraße 74, 01307 Dresden, Germany. E-mail: catharina.schuetz@tu-dresden.de

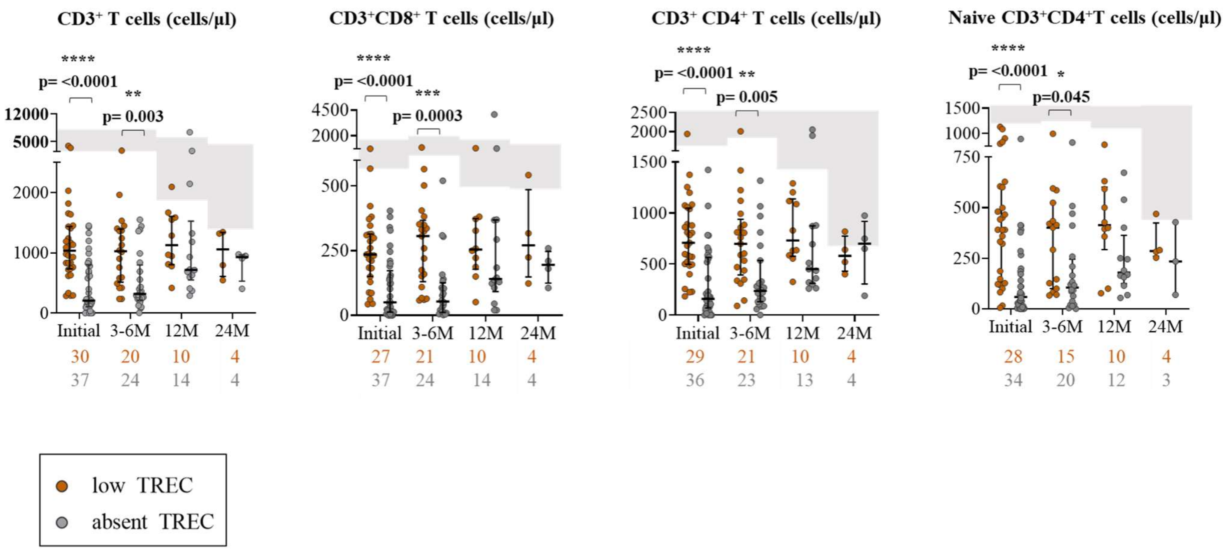


**Fig. S1** Cell counts of patients with low TREC-results (brown) and absent TREC-results (grey) on newborn screening at different time points: initial screening at 3-6, 12 and 24 months of age. Approximate age-dependent paediatric reference values according to Shearer *et al*. (2003) in grey. Cell counts of total lymphocytes, total T cells, CD8+ and CD4+ T cells and naïve CD4+ T cell, as well as available patient numbers at each time point (below the graphs). Cell counts after transplantation not included. Black bars indicate median and interquartile range. Student's t-test was applied to assess significance
